# Decisions at the Brink: Locomotor Experience Affects Infants’ Use of Social Information on an Adjustable Drop-off

**DOI:** 10.3389/fpsyg.2016.00797

**Published:** 2016-06-03

**Authors:** Lana B. Karasik, Catherine S. Tamis-LeMonda, Karen E. Adolph

**Affiliations:** ^1^Department of Psychology, College of Staten Island and Graduate Center, City University of New York, New YorkNY, USA; ^2^Department of Applied Psychology, New York University, New YorkNY, USA; ^3^Department of Psychology, New York University, New YorkNY, USA

**Keywords:** infant locomotion, social cognition, perceptual exploration, crawling, walking

## Abstract

How do infants decide what to do at the brink of a precipice? Infants could use two sources of information to guide their actions: perceptual information generated by their own exploratory activity and social information offered by their caregivers. The current study investigated the role of locomotor experience in using social information—both encouragement and discouragement—for descending drop-offs. Mothers of 30 infants (experienced 12-month-old crawlers, novice 12-month-old walkers, and experienced 18-month-old walkers) encouraged and discouraged descent on a gradation of drop-offs (safe “steps” and risky “cliffs”). Novice walkers descended more frequently than experienced crawlers and walkers and fell while attempting to walk over impossibly high cliffs. All infants showed evidence of integrating perceptual and social information, but locomotor experience affected infants’ use of social messages, especially on risky drop-offs. Experienced crawlers and walkers selectively deferred to social information when perceptual information is ambiguous. In contrast, novice walkers took mothers’ advice inconsistently and only at extreme drop-offs.

## Introduction

How do infants appraise the situation while peering over the top of a staircase as their mother screams for them to stop? How do they decide what to do when perched at the top of a playground slide as their mother beckons with open arms from the bottom? In such potentially risky situations, two sources of information are available to guide motor action—perceptual information generated from infants’ own exploratory activity and social information offered by caregivers (Tamis-LeMonda et al., 2008). Both types of information can convey to infants whether an action is possible or should be avoided (Franchak and Adolph, 2014). Thus, when infants are uncertain about how to act based on perceptual information, caregivers may know best how to respond. Picture these everyday examples: after a hard fall, infants often look to their mothers to decide whether to cry or to keep going, and their reactions may depend on mothers’ frightened gasp or reassuring smile. Other times, infants explore forbidden situations despite mothers’ discouragement: they dig into the bowl of dog food, completely ignoring mothers’ prohibition to stop (Tamis-LeMonda et al., 2007).

### Infants’ Use of Social Information in Risky Situations

In everyday situations, perceptual and social information are often simultaneously available, and infants must evaluate the credibility of each source when deciding how to act. Infants should place more weight on perceptual information when it clearly specifies that an action is safe or risky, possible or impossible. But when perceptual information leaves infants uncertain, they should be more likely to defer to the social information. Indeed, according to the classic definition of “social referencing” infants should use social information only in situations of ambiguity (Feinman et al., 1992; Baldwin and Moses, 1996).

Infants’ use of social information for guiding locomotion is most famously illustrated in a study of infants on the “visual cliff” (Sorce et al., 1985). Twelve-month-old crawling infants used mothers’ facial expressions to determine whether to cross a 30-cm apparent drop-off. The risk of the drop-off was considered “ambiguous” because in pilot work, infants paused at the edge and looked to their mothers. The drop-off was apparent, not real, because safety glass over the surface protected infants from falling. During test trials, mothers stood at the far side of the apparatus and posed “happy” or “fearful” facial expressions. Most infants (74%) crossed the visual cliff when mothers displayed a happy face but none crossed when mothers displayed fear.

Although the study is widely cited, several problems undermine interpretations about infants’ ability to weigh and integrate perceptual and social information. The visual cliff was restricted to only one drop-off height, 30 cm. Thus, we cannot know how infants would respond to drop-offs that vary in apparent risk. It is unlikely that the 30-cm drop-off was truly ambiguous for most infants. Recent work shows that the boundary between safe and risky drop-off heights varies widely (from 6 to 23 cm) among 12-month-old crawlers, and 30 cm is risky for most infants, not ambiguous (Kretch and Adolph, 2013). In fact, 12-month-olds treat an actual 30-cm drop-off as risky and avoid crawling over the edge. In addition, infants on the visual cliff were tested in only one trial, precluding within-subject comparisons of their use of both positive and negative messages. Moreover, infants were exposed to static facial expressions, which may be unnatural and distressing to them (Tronick et al., 1978), perhaps explaining the 40% attrition rate in both conditions (Sorce et al., 1985). Relatedly, many infants did not benefit from the social message at all; mothers were not allowed to use words, sounds, or gestures, and most infants did not look to mothers before crossing. Indeed, a subsequent study showed that mothers’ vocal messages are especially important for infants’ crossing behavior (Vaish and Striano, 2004).

A suite of issues concerns the safety glass. The glass surface looks risky but feels safe and infants quickly figure out that the glass is perfectly traversable. Accordingly, they can be tested in only one trial and findings are reported in terms of the proportion of infants who cross. More troubling, the safety glass is too forgiving of infants’ movements, leading researchers to misinterpret infants’ decisions. In the fear condition, 65% of infants (11 of 17) crossed the brink and retreated, but ultimately none crawled to their mothers at the far side of the apparatus, leading to the interpretation that mothers’ fearful facial expression caused infants to avoid crossing. However, without the safety glass, the “partial crossers” in the fear condition would have fallen as soon as they placed their hands over the brink and thus considered to be “crossers” not avoiders. Moreover, the safety glass prevents infants from using alternative crossing strategies (e.g., backing down feet first), which would provide additional evidence of their integration of perceptual and social information.

Finally, only one group was tested—12-month-old crawlers—precluding examination of the role of locomotor experience. Recent work shows that differences in infants’ locomotor experience affect their ability to distinguish safe from risky drop-offs. In particular, 12-month-old crawlers are likely to have several months of crawling experience and 12-month-old walkers are likely to have just begun walking. Although, one study found no difference between 12-month-old experienced crawlers and 12-month-old novice walkers in attempts to cross a visual cliff (Witherington et al., 2005), several studies found robust differences between experienced crawlers and novice walkers on real drop-offs and slopes (Adolph, 1997; Adolph et al., 2008; Kretch and Adolph, 2013). Specifically, 12-month-old experienced crawlers, like 18-month-old experienced walkers, accurately judged which drop-offs and slopes were safe for crawling and walking (Adolph, 1997; Adolph et al., 2008; Kretch and Adolph, 2013). But, 12-month-old novice walkers did not, suggesting that experience, not age, is the critical factor for distinguishing safe from risky drop-offs.

**FIGURE 1:**
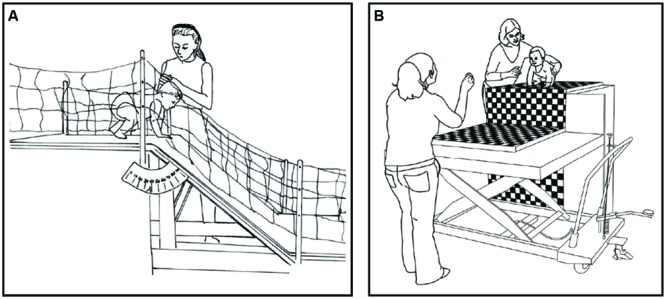
**(A)** Adjustable slope apparatus used in previous work. **(B)** Adjustable drop-off apparatus. Infants began in a prone or upright position on the starting platform. An experimenter followed alongside infants to ensure their safety. The landing platform adjusted in 1-cm increments from 0 to 90 cm. In blocked test trials, mothers encouraged and discouraged infants’ descent; they stood at the landing platform.

Use of an adjustable slope paradigm (**Figure 1A**) circumvented many of the methodological issues with the visual cliff (Adolph et al., 2008, 2010, 2014; Tamis-LeMonda et al., 2008). Whereas the visual cliff paradigm assumed a one-size-fits-all definition of risk, in the slope paradigm, risk was normalized to each infant’s ability by using a psychophysical procedure to estimate the steepest slope infants could crawl or walk down successfully. By definition, this “borderline” slope marked the boundary between slopes that were safe (shallower than borderline) and slopes that were risky (steeper than borderline). Infants were tested across a range of safe and risky increments, rather than at the two risk settings of shallow and deep, because the apparatus varied in 2° increments from 0 to 50°. Whereas on the visual cliff infants received only one test trial with a posed facial expression, on slopes infants were tested over multiple trials at safe, risky, and borderline slopes, and mothers delivered natural dynamic (rather than posed) encouraging and discouraging messages (Karasik et al., 2008). Instead of safety glass as on the visual cliff, in the slopes paradigm an experimenter followed alongside infants to ensure their safety. Previous work showed that infants do not rely on the experimenter for rescue over multiple trials, because when tested longitudinally, infants become more cautious, not more reckless (Adolph, 1997). Thus, it is possible to collect dozens of trials per baby and to report findings in terms of the average proportion of trials on which infants crawl or walk (Adolph, 2000; Adolph and Avolio, 2000). Finally, the slope paradigm compared 12-month-old experienced crawlers, 12-month-old novice walkers, and 18-month-old experienced walkers, rather than one 12-month-old crawler group, enabling assessment of age and experience on infants’ decisions. By addressing many of the methodological limitations of the visual cliff, studies using the slope paradigm highlighted infants’ selective use of social information. Experienced 18-month-old walking infants used mothers’ social information only at the borderline slope: they walked when their mothers encouraged and not when their mothers discouraged (Tamis-LeMonda et al., 2008). But on safe and risky slopes, where the perceptual information was clear, 18-month-olds ignored the social information: they walked down safe slopes and refused to walk down risky ones, regardless of mothers’ messages (Tamis-LeMonda et al., 2008). Moreover, when the region of uncertainty was experimentally manipulated—by dressing 18-month-olds in slippery Teflon-soled shoes—infants correspondingly treated shallow slopes (0–15°) as uncertain and deferred to mothers’ social messages (Adolph et al., 2010).

Twelve-month-olds presented a very different picture. Experienced 12-month-old crawlers deferred to mothers’ messages only on safe slopes and they refused to crawl down borderline and risky slopes regardless of whether mothers encouraged or discouraged (Adolph et al., 2008). Novice 12-month-old walking infants attempted both safe and risky slopes in the encouraging condition; they walked repeatedly over the brink of an impossibly steep 50° slope on 75% of trials. In the discouraging condition, they were less likely to attempt the 50° slope, but they still plunged over the brink on more than 50% of trials.

The slope studies are not definitive, however, because slopes and drop-offs present infants with different types of challenges; notably a slope has a gradual drop-off whereas a drop-off has an abrupt discontinuity. These structural differences are accompanied by differences in visual and haptic information as infants explore the obstacle and by differences in the affordances for descent (generally, slopes are easier to descend than a sheer drop-off with the same vertical height). Recent work partially addressed methodological differences between the visual cliff and adjustable slopes by testing infants at the edge of a *real*, adjustable drop-off (Kretch and Adolph, 2013). Like the adjustable slope, the drop-off varies in 1-cm increments from 0 to 90 cm. The largest drop-off was an actual cliff, higher than infants’ standing height. Mothers and an experimenter encouraged infants to crawl or walk using words, gestures, and facial expressions, and offering toys and snacks as incentives. Consistent with previous work on slopes, experienced 12-month-old crawlers and experienced 18-month-old walkers made accurate decisions: they always crossed safe drop-offs, but on risky drop-offs they ignored the melee of encouragement and refused to crawl or walk. In contrast, novice 12-month-old walkers appeared largely oblivious to risk. They walked over safe drop-offs, but they also walked over impossibly risky ones; they walked over the brink of the 90-cm cliff on 50% of trials.

However, Kretch and Adolph’s (2013) adjustable drop-off could not address the question of whether infants weigh and integrate social and perceptual information because the social information was constant: the adults always beckoned infants to crawl or walk down, preventing comparison of infant decisions when encouraged and discouraged. Moreover, to ask whether infants differentially use mothers’ social messages as in the slope studies, the social information must come only from the mother, without use of external incentives. But the infants in Kretch and Adolph’s study received a double dose of encouraging social information—from both mother and experimenter—and the adults used toys and snacks as lures.

Previous work prevents comparison of infants’ interpretation of risk on an adjustable drop-off versus slope because the two apparatuses present different obstacles and the social information was only encouraging. On the cliff apparatus, a 50-cm drop-off was impossible to descend by crawling or walking, but on the slope apparatus, the same drop-off height was mediated by a 36° slope between starting and landing platform, which many infants could safely crawl or walk down (Adolph, 1997; Tamis-LeMonda et al., 2008; Kretch and Adolph, 2013). Infants may view the gradual drop-off of a steep slope to be more conducive to alternative descent strategies compared with the abrupt drop-off of a cliff. For example, in response to encouragement on 50° slopes, 18-month-olds used alternative descent strategies (sliding, backing) on approximately 80% of trials, but on a 90-cm drop-off, they used alternative strategies to descent on only 41% of trials. Given the perceptual differences of the drop-off, differential social information may influence infants’ use of alternative descent strategies.

### Current Study

The current study focused on infants’ behavior at the brink of an adjustable drop-off. On some trials, the drop-off was safe—essentially a “step”—and on other trials, the drop-off was risky—a “cliff.” Errors in motor decisions were met with real consequences; one wrong move and infants fell into an experimenter’s arms. Similar to the slope, the drop-off apparatus challenged infants with parametric variations in height, but the drop-off presented an abrupt discontinuity in the surface. A sheer drop-off may influence infants’ interpretations of mothers’ encouraging and discouraging social messages and thereby affect their decisions about whether to crawl, walk, select an alternative descent strategy, or avoid going.

The primary aim was to test whether locomotor experience and age affect infants’ use of social information at the edge of a drop-off. As in the slopes studies (Adolph et al., 2008, 2010; Tamis-LeMonda et al., 2008), we tested three groups of infants: 12-month-old experienced crawlers, 12-month-old novice walkers, and 18-month-old experienced walkers. First, we used the psychophysical staircase procedure developed in earlier work (Adolph, 1995, 2000) to identify the borderline drop-off—the largest drop-off infants could descend in their typical crawling or walking posture. The borderline increment delineated the boundary between safe and risky drop-offs for each infant. Then, in two blocked conditions over multiple trials on safe, borderline, and risky drop-offs, mothers encouraged and discouraged their infants. Mothers were free to use facial expressions, vocal intonations, words, and gestures to provide their social message. Because the focus was on infants’ use of social information, mothers were not allowed to use toys or food as incentives. Encouragement and discouragement were unsolicited—mothers provided social messages at the start of each trial, regardless of whether infants looked at them first. The primary outcome measure was whether infants attempted to crawl or walk. In addition, we asked whether social information influenced alternative descent strategies such as scooting in a sitting posture or backing down feet first and infants’ latency to make their decisions. As in our earlier work, we used a within- rather than between-subjects design to compare infants’ responses to varying messages across multiple trials.

Based on previous work (Adolph et al., 2008; Tamis-LeMonda et al., 2008; Kretch and Adolph, 2013), we expected experienced crawlers and walkers to rely largely on perceptual information at safe and risky drop-offs, but to defer to mothers’ advice at borderline increments. Most interesting are infants’ responses in the discouraging condition. If experienced infants respond to mothers’ discouragement in the same way as they had on slopes, on safe drop-offs, they should ignore mothers’ message and attempt to crawl and walk down. But on risky drop-offs, experienced crawlers and walkers should flatly refuse their typical method of descent and novice walkers should attempt less frequently, but fall repeatedly nonetheless. Notably, at borderline increments, infants’ responses to mothers’ encouragement and discouragement should diverge: infants should decrease attempts to walk or crawl when mothers discourage and increase attempts to walk or crawl when mothers encourage.

In contrast, we expected novice walkers to be less adaptive in the use of perceptual information, and to walk over the edge of both safe and risky drop-offs, repeatedly falling on risky drop-offs. Further, although we expected novice infants to be swayed by mothers’ messages—increasing their attempts to walk in the encouraging condition and decreasing attempts in the discouraging condition—they should not integrate social and perceptual information. Unlike experienced crawlers and walkers, novices will not selectively use social information at borderline increments.

Due to differences in the slope and cliff tasks, it is possible that infants will show slightly shifted patterns of responding in the context of drop-offs. If infants view an abrupt drop-off with more wariness than a slope, then they should show more reticence to crawl or walk on safe and borderline increments when mothers discourage. In either case, increased latency on risky drop offs would indicate that infants attended to the message, regardless of whether they followed mothers’ advice. On trials where infants refuse to crawl and walk, the content of mothers’ message may also affect infants’ selection of alternative descent strategies. They may interpret encouragement to crawl or walk more broadly as encouragement to descend and/or discouragement to crawl or walk as a suggestion to use an alternative method of locomotion.

A secondary aim was to confirm that encouraging social information was not the critical determinant in differences between 12-month-old novice walkers and 12-month-old experienced crawlers in previous work with the adjustable drop-off (Kretch and Adolph, 2013). We know from previous work on slopes that novice 12-month-old walkers attempt to walk down risky slopes at high rates regardless of whether mothers encourage or discourage, although discouragement leads to decreased attempts to walk at the steepest increment (Adolph et al., 2008). Given the perceptual differences between drop-offs and slopes, it was important to determine whether 12-month-old novice walkers would still be inclined to walk down large drop-offs even when discouraged.

## Materials and Methods

### Participants

Thirty healthy, full-term infants and their mothers were recruited from mailing lists, referrals, and local hospitals. Infants were 12 months old (±1 week) or 18 months old (±1 week). Most families were White and middle-class, and all were college educated. All mothers (*M*age = 34.00 years, *SD* = 4.98) spoke English as their primary language at home and identified as their infants’ primary caregiver. Families received souvenirs of participation. Data from five infants were excluded due to fussiness. The overall attrition rate (5/35 = 14%) is similar to our previous work varying social messages on slopes (e.g., Adolph et al., 2008; Tamis-LeMonda et al., 2008).

Eleven 12-month-olds were crawlers but not yet walkers (five girls, six boys); 10 12-month-olds were walkers (five girls, five boys); and nine 18-month-olds were walkers (five boys, four girls). The 12- and 18-month-old walkers could also crawl, but no longer did so habitually. Mothers reported infants’ locomotor experience during a structured interview (as in Adolph et al., 2011) using baby books and calendars. Crawling experience dated from the first day infants crawled 10 feet continuously until the test day and walking experience dated from infants’ first success at walking 10 feet continuously without support. Crawling experience was equivalent in the two groups of 12-month-old: *M* = 4.26 months (*SD* = 1.70) for the crawlers and *M* = 3.48 months (*SD* = 1.22) for the walkers, *p* > 0.10. However, the 12-month-old crawlers had more crawling experience (*M* = 4.26 months, *SD* = 1.70) than the 12-month-old walkers had walking experience (*M* = 1.41 months, *SD* = 0.85); and comparable crawling experience to the 18-month-olds’ walking experience (*M* = 5.40 months, *SD* = 2.00). Thus, the 12-month-old crawlers and 18-month-old walkers were the “experienced” groups, and the 12-month-old walkers were “novices.” *Post hoc* analyses revealed differences in locomotor experience only between the experienced and novice groups, *F*(2,28) = 16.66, *p* < 0.001.

### Drop-off Apparatus

Wooden starting (90 cm wide × 90 cm long) and landing (120 cm wide × 120 cm long) platforms placed side by side were lined with high-density foam for safety and covered with a black-and-white checkerboard pattern for visual salience (**Figure 1B**). The starting platform stood at a permanent height (120 cm) and the landing platform was affixed to a hydraulic lift operated by a push pedal. One assistant adjusted the height of the landing platform in 1-cm increments to create drop-offs varying from 0 to 90 cm. A stationary camera recorded the height of the drop-off from a ruler at the side of the platform. A second stationary camera positioned opposite to the landing platform recorded mothers’ behaviors. A second assistant operated a panning camera to record infants from a side view.

### Procedure

Each session was divided into two parts: a psychophysical procedure to estimate a borderline drop-off for the individual infant and a probe procedure to determine infants’ use of social information. During the psychophysical procedure, mothers and an assistant stood at the end of the landing platform and encouraged infants to descend using toys and snacks as incentives. During the probe trials, mothers encouraged or discouraged infants to crawl/walk with no help from an assistant and no use of lures. Sessions lasted approximately 90 min. At the start of each trial, the experimenter placed infants on the starting platform on hands and knees (for crawlers) or standing upright (for walkers) and followed alongside infants to ensure their safety. Trials lasted 30 s or until infants attempted descent, whichever came first.

To estimate each infant’s borderline drop-off, we used the psychophysical staircase procedure developed in earlier work (e.g., Adolph, 1995, 2000; Kretch and Adolph, 2013). To ensure that infants were comfortable crawling or walking over the apparatus, they first received four warm-up trials on the 0-cm drop-off. Then infants were encouraged to descend a 1-cm baseline drop-off. After successful trials (infants crawled or walked safely), drop-off height was increased by 3 cm. After two unsuccessful trials (infants tried to crawl or walk but fell or infants refused to crawl or walk by avoiding descent or using an alternative descent method), the drop-off was decreased by 2 cm. After unsuccessful trials, 1-cm baselines were presented to renew infants’ motivation. This up–down procedure continued until converging on a *borderline* drop-off with a 67% criterion—the largest drop-off with at least two out of three successful trials and at least two out of three unsuccessful trials at the next 1-, 2-, and 3-cm drop-offs. Infants contributed *M* = 44.14 trials (range = 27–64) in the psychophysical part of the experiment.

The actual test of infants’ use of social information was assessed during probe trials at five risk increments blocked into two social conditions—encouraging and discouraging—with condition order counterbalanced across gender. Five risk increments were presented in four quasi-random orders within each social condition: 1-cm drop-off, *borderline, safe* (-6 cm from the borderline increment), *risky* (+6 cm from the borderline increment), and at 90-cm drop-off. Infants were tested at the absolute 1- and 90-cm drop-offs to compare all infants on the same increments. After collecting the set of probes for each encouragement and discouragement block, the experimenter attempted to run each set again. Infants contributed at least 1 trial at each drop-off in each social condition. On average, infants received 6.67 encouragement trials (*SD* = 1.42) and 6.77 discouragement trials (*SD* = 1.33).

Mothers were allowed to use any words, gestures, and facial expressions that seemed natural to them to convey encouragement and discouragement to their infants. They were instructed to get their infants to crawl/walk down or prevent their infants from crawling/walking down while disregarding the size of the drop-off. Previous work verified that mothers’ encouragement and discouragement varied by condition but did not vary by risk level (Karasik et al., 2008; Tamis-LeMonda et al., 2008). Mothers stood alone at the far end of the apparatus at infants’ eye level (**Figure 1B**). Toys and snacks were removed. An assistant rang a bell to signal to mothers to begin encouraging or discouraging infants while the experimenter held infants on the starting platform for 2 s to ensure that infants received the social message on each trial. After infants were released, mothers continued to deliver their messages.

### Data Coding

Coders scored the videos using a computerized video coding system, Datavyu^1^. Infants’ motor *decisions* were coded as success (crawled or walked safely), failure (attempted to crawl or walk but fell), or refusal (avoided going or used an alternative descent strategy). For refusal trials, the coders scored whether infants backed down on their bellies with feet toward the landing platform, scooted down on their bottoms in a sitting position, or crawled (for walkers). Infants’ *latency* to descend was coded from the moment the experimenter released infants on the starting platform until infants initiated descent. Latency included the time that infants explored drop-offs, but the time required for infants to get into their final descent position was subtracted. Thus, latency could range from 0 (immediate decision) to 30 s (avoid descent) and infants were not penalized for selecting an alternative descent strategy.

For each probe trial, coders scored the content of mothers’ verbal messages in the two social conditions as statements about the *target action* (e.g., “Walk over here,” “No walking”), *general directives* that affirmed or prohibited movement without providing specific information about locomotor posture (e.g., “Come on,” “No, stay there”), *alternative strategies* for descent (“Back down”), and *distractors* (e.g., “Let’s dance”).

A primary coder scored 100% of the data, and a second coder scored 25–30% of each infant’s data to ensure inter-rater reliability. Percent agreement was 95–99% for all categorical variables (κ = 0.95 to 0.97); the correlation coefficient for latency was *r*(117) = 0.99.

## Results

**FIGURE 2:**
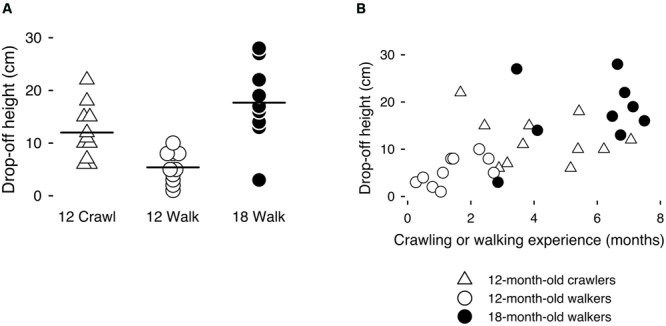
**(A)** Borderline drop-offs at the limit of infants’ crawling and walking ability for 12-month-old crawlers, 12-month-old walkers, and 18-month-old walkers. Each symbol represents one infant; solid lines represent group means. **(B)** Relations between posture-specific locomotor experience (months since crawling onset for crawlers, months since walking onset for walkers) and individual differences in borderline drop-offs, at the limit of infants’ abilities.

A wide range in infants’ borderline drop-offs (**Figure 2A**) within and across the locomotor groups confirmed the need to individualize risk level for each infant. On average, borderline drop-offs were larger for the experienced 12-month-old crawlers (*M* = 12.00, range = 6–22 cm) and 18-month-old walkers (*M* = 16.10 cm, range = 2–28 cm) than for the novice 12-month-old walkers (*M* = 5.40, range = 1–10 cm), *F*(2,28) = 7.94, *p* < 0.05, η^2^ = 0.42; *post hoc* tests revealed differences only between the novice and experienced groups. Infants with more crawling experience (for crawlers) and walking experience (for walkers) had larger borderline drop-offs, providing verification of estimates of infants’ abilities, *pr*(27) = 0.38, *p* < 0.05, controlling for age (**Figure 2B**). Infants’ borderline drop-offs were comparable to previous work (Kretch and Adolph, 2013).

Mothers’ messages aligned with the social condition as in previous work (Karasik et al., 2008; Tamis-LeMonda et al., 2008). They encouraged or discouraged on every trial as instructed, and they showed no difference in their overall use of target actions, general directives, and distractors across conditions or risk levels (*p*s > 0.05). Mothers *never* mentioned alternative descent strategies. On most trials, mothers used general directives (“Come on,” “No, stop,” 72.93%). They occasionally accompanied general directives with target actions (“Crawl down,” “No walking,” 11.87%) or with distractors (“Clap your hands,” 7.79%). They rarely used only target actions (2.04%), only distractors (4.08%), or general, target, and distractors within a single trial (1.30%).

### Decisions to Crawl and Walk

**FIGURE 3:**
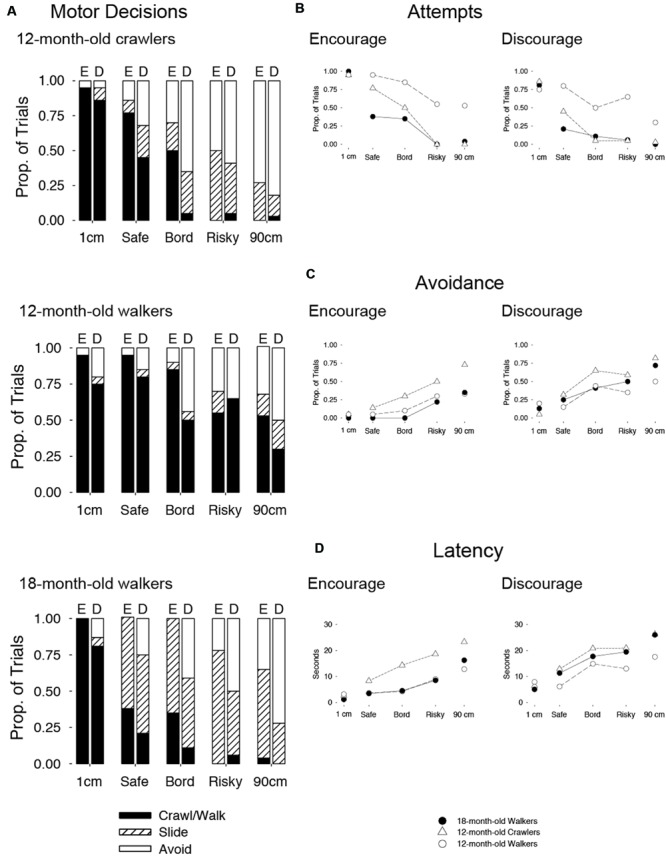
**(A)** Stacked bar graphs show the average proportion of trials in, which 12-month-old experienced crawlers, 12-month-old novice walkers, and 18-month-old experienced walkers attempted to crawl or walk (black bars), used alternative descent strategies (striped bars), or avoided descent (white bars) in the encouraging “E” and discouraging “D” conditions. The x-axis is labeled with five drop-off increments in order of increasing risk: 1-cm, safe (-6-cm smaller than infants’ borderline drop-off), borderline drop-off, risky (+6-cm larger than infants’ borderline drop-off), and 90-cm drop-off. Line graphs show **(B)** proportion of trials in which infants attempted to crawl or walk and **(C)** avoid. **(D)** Latency (seconds) graphs show hesitation on the starting platform in each locomotor group.

**Figures 3A,B** shows infants’ decisions to descend the drop-offs. Regardless of mothers’ social message, infants in all three locomotor groups crawled or walked over the 1-cm drop-off (see large black bars in **Figure 3A** and line graphs in **Figure 3B**). But on other drop-offs, responses diverged depending on infants’ locomotor group and social condition. The experienced crawlers and walkers rarely crawled or walked over the risky (+6 cm) and 90-cm drop-offs, regardless of mothers’ message (see tiny black bars in **Figure 3A**). In contrast, the novice walkers frequently attempted the risky and 90-cm drop-offs in both social conditions, requiring rescue by the experimenter (see black bars in **Figure 3A**). Indeed, locomotor experience (crawling for crawlers and walking for walkers) was negatively correlated with attempt rates on risky and 90-cm drop-offs combined, *pr*(28) = -0.51, *p* < 0.01, controlling for age.

To formally compare infants’ attempts to crawl or walk in the three locomotor groups and two social conditions across the five risk levels, we analyzed infants’ decisions at the trial level. Across the sample, infants received a total of 403 probe trials. Because infants contributed 1–2 trials per risk level per condition, a repeated measures ANOVA was not appropriate. Instead, we used a mixed model analysis with the total number of useable trials as a scale weight factor. Best model fit was determined based on goodness of fit QIC information. This analysis yielded main effects of locomotor group [*F*(2,29) = 11.56, *p* < 0.001], condition [*F*(1,49) = 16.18, *p* < 0.001], and risk [*F*(4,68) = 50.86, *p* < 0.001], a condition by risk interaction [*F*(8,67) = 5.80, *p* < 0.001], and a three-way interaction [*F*(12,62) = 3.02, *p* < 0.01]. Most notable is the three-way interaction, suggesting that locomotor experience had a differential effect on infants’ use of social information to crawl and walk at different risk levels. We used Sidak corrections for multiple comparisons (*p*s < 0.05).

The *post hoc* tests confirmed differences between social conditions for the 18-month-old walkers at the borderline drop-off; the 12-month-old crawlers at the safe and borderline drop-offs; and the 12-month-old walkers at the borderline and 90-cm drop-offs. At these increments, infants were more likely to crawl or walk when mothers encouraged than when they discouraged (compare height of black bars across social conditions at these increments). In line with the expectation that experienced infants would selectively use social information, 18-month-olds were more likely to walk when mothers encouraged than when they discouraged but only at the borderline drop-off (*p* < 0.05, *d* = 0.42). Experienced 12-month-old crawlers were more likely to crawl when mothers encouraged than when they discouraged at safe (*p* < 0.01, *d* = 0.53) and borderline drop-offs (*p* < 0.001, *d* = 0.76). Novice 12-month-old walkers were more likely to walk when mothers encouraged than when they discouraged at borderline (*p* < 0.01, *d* = 0.54) and 90-cm drop-offs (*p* < 0.05, *d* = 0.39).

### Avoidance and Alternative Strategies

Social information and risk level also affected infants’ behavior on trials where they did not crawl or walk. As shown by the white bars (**Figure 3A**) and “avoid” line graphs in **Figure 3C**, infants showed more avoidance in the discouraging condition and increased avoidance with risk. A mixed linear model confirmed main effects of condition [*F*(1,47) = 25.24, *p* < 0.001, *d* = 0.92] and risk [*F*(4,63) = 17.95, *p* < 0.001, *d*s = 0.28 to 0.70].

However, avoidance did not merely mirror attempts to crawl or walk. Rather, infants often used alternative descent strategies on trials where they refused to crawl or walk (striped bars in **Figure 3**). Most of the 12-month-old crawlers (8 of 11 infants) executed an alternative strategy on at least one trial and when they did they backed down feet first (11.69% of trials) or lowered themselves over the drop-off by kneeling (10.39% of trials). Only 4 of 10 12-month-old walkers used alternatives, either backing (4.65% of trials) or sitting (3.86% of trials). Notably, the novice walkers almost never reverted to crawling (2.32% of trials). All 18-month-old displayed alternative methods of descent by either sitting (26.67% of trials) or backing (20.00% of trials), and almost never by crawling (0.83% of trials).

A mixed linear model on frequency of alternative descent strategies revealed main effects of locomotor group [*F*(2,30) = 7.80, *p* < 0.01] and risk [*F*(4,69) = 9.00, *p* < 0.001], two-way interactions between locomotor group and condition [*F*(2,34) = 3.48, *p* < 0.05] and locomotor group and risk [*F*(8,70) = 3.35, *p* < 0.01], and a three-way interaction between factors [*F*(12,52) = 2.38, *p* < 0.05]. *Post hoc* analyses confirmed these effects. Overall, the 18-month-olds used alternative strategies (48.33% of trials) more frequently than did the other locomotor groups, especially 12-month-old walkers (10.85% of trials, *p* < 0.01, *d* = 0.50). Moreover, 18-month-olds used alternative strategies more frequently than they avoided especially, on risky and 90-cm drop-offs in the encouraging compared to discouraging condition (*p* < 0.05, *d* = 0.64). The experienced 12-month-old crawlers used alternative strategies (25.32% of trials) less frequently than they avoided (44.81% of trials); alternatives were more frequent at the risky drop-offs (*p* < 0.05, *d* = 0.66), and did not depend on the social condition. The novice 12-month-old walkers used alternatives sporadically (10.85% of trials) and at times avoided (27.91% of trials).

### Latency

Latency (line graphs in **Figure 3D**) to descend increased when mothers discouraged and latency increased with risk level. Increased latency did not merely reflect avoidance; similar patterns emerged when we removed trials in which infants avoided descent. A mixed linear model confirmed main effects only for condition [*F*(1,42) = 32.70, *p* < 0.001, *d* = 1.88] and risk [*F*(4,58) = 24.54, *p* < 0.001, *d*s = 2.11 to 3.00]. In other words, mothers’ discouragement held infants for a few seconds longer at the brink of the drop-off, even on drop-offs where infants decided to descend.

## Discussion

We addressed the role of locomotor experience and age in infants’ use of social information at the brink of drop-offs that varied in risk. Similar to Sorce et al.’s (1985) classic social referencing study on the visual cliff, mothers encouraged and discouraged without toys/snacks so that social information was carried solely in mothers’ communication. But, unlike the classic study, mothers’ mode of communication was unrestricted; they used their face, voice, words, and gestures however felt natural; and mothers delivered the social information regardless of whether infants looked at them first. Our central goal was to ask whether experienced crawlers and walkers, but not novice walkers, integrate perceptual and social information on drop-offs, in line with previous research on slopes (Adolph et al., 2008, 2010; Tamis-LeMonda et al., 2008). We anticipated possible shifts in the pattern of perceptual-social integration by experienced infants due to structural and perceptual differences between slopes and sheer drop-offs. Infants might view a drop-off with more wariness than a slope, and thus be especially receptive to social information when mothers discourage. Finally, we asked whether 12-month-old walkers would avoid walking down cliffs when mothers discouraged, or instead continue to walk over large drop-offs—a sign of their inability to gage affordances for locomotion in their new posture.

We found that locomotor experience and age affected infants’ use of social information on drop-offs: experienced infants selectively used social information to guide their decisions but ignored encouragement on large drop-offs where perceptual information specified risk. Discouragement resulted in decreased attempts to walk for 12-month-old walkers, but they still displayed poor decisions. Thus, this finding confirmed that encouraging social messages were not the critical determinant in differences between 12-month-old experienced crawlers and 12-month-old novice walkers in previous work with adjustable drop-offs and slopes (Kretch and Adolph, 2013). We describe each set of findings below.

### Infants’ Use of Social Information on Drop-offs

In line with our previous work in the slope paradigm, infants in all three locomotor groups used mothers’ social messages in their decisions to descend drop-offs. But, infants’ attempt rates on drop-offs were lower compared with attempt rates on slopes (Adolph et al., 2008; Tamis-LeMonda et al., 2008). Possibly, the discontinuous surface of the drop-off presents different challenges and perceptual information such that experienced crawlers and walkers treated the drop-off with more caution.

Locomotor experience played an important role in determining at which risk levels social information swayed infants’ decisions to attempt descent and to what extent. Experienced crawlers and walkers selectively ignored mothers’ social information on small and large drop-offs: they crawled and walked on the smallest 1-cm drop-off and refused to attempt their typical method of locomotion on the risky (+6 cm) and 90-cm drop-offs, regardless of mothers’ messages. As in previous work (Tamis-LeMonda et al., 2008), experienced 18-month-old walkers were the most discerning. They deferred to mothers’ message only at the borderline drop-off, where they attempted to walk when mothers encouraged but not when mothers discouraged. Also replicating previous work (Adolph et al., 2008), the experienced 12-month-old crawlers were less precise; they deferred to social information at both borderline and safe drop-offs. Finally, as in previous work (Adolph et al., 2008), the novice 12-month-old walkers only benefitted from social information at the most extreme 90-cm drop-off. Although, novice walkers also showed differential attempt rates by social condition at the borderline increment, their attempts did not differ by social condition at the riskier +6-cm drop-off, indicating that their use of social information was inconsistent.

One explanation for these findings is that infants considered mothers’ messages at every drop-off, but at particular drop-offs gave more weight to the perceptual information generated by their own exploratory activity. In this case, latency to descend would reflect both infants’ attention to mothers’ message and to the consequences of their own exploratory activity and we should see longer latencies on trials when mothers discouraged across risk levels. An alternative explanation is that infants attended to the social information only at particular drop-offs; that is, they first gauged the slope based on perceptual information and attended to social information only after determining that the perceptual information was insufficient. In this case, latency would reflect only risk level, and we should see no differences by social condition or an interaction between social condition and risk level. The latency data support the former explanation (**Figure 3D**). Infants in all three groups hesitated almost twice as long in the discouraging condition compared to the encouraging condition and there was no interaction between locomotor experience group and social condition. Together, the attempt and latency data suggest that infants were attentive to mothers’ messages, yet still sometimes chose to disregard them.

### Infants Generate Unique Solutions for Descending Drop-offs

Infants sometimes used alternative strategies to descend drop-offs. Did they do so spontaneously or in response to recommendations by their mothers? Because mothers did not follow scripted messages, we were able to examine how infants descended based on mothers’ natural encouragement and discouragement. We found that mothers always encouraged and discouraged for the appropriate condition, usually with general directives to come down or stay put. Yet, they never advised infants to descend using an alternative method (e.g., back down). Thus, infants’ use of alternative descent strategies was wholly their own idea. Alternative strategies were more common in the encouraging condition compared with the discouraging condition, on larger drop-offs compared with smaller ones, and in the 18-month-olds compared with the 12-month-olds. Similar descent strategies were available to infants: crawlers typically backed and kneeled and walkers backed and scooted. Presumably, the novice 12-month-old walkers displayed few alternative strategies because they rarely refused to walk. Their avoidance rate matched that of 18-month-old walkers. But, experienced 18-month-walkers chose alternatives rather than avoid. The experienced 12-month-old crawlers knew better than to crawl over the brink. Likely, they displayed less frequent use of alternative descent methods because of the difficulty in executing them. Backing, for example, requires an initial detour away from the goal, and then moving backward without visual guidance of the destination—difficult feats for young infants (McGraw, 1935; Adolph, 1997).

Locomotor experience and age explained infants’ use of alternative descent methods across risk and social condition. On risky trials when mothers encouraged using general directives, experienced 12-month-old crawlers and 18-month-old walkers complied with the message by finding an appropriate way to come down. Accordingly, on most trials, experienced infants switched from their starting crawling or walking posture to sliding. In contrast, 12-month-old novices walked when mothers encouraged with general directives. Thus, 12-month-old walkers responded to the message by continuing in their starting posture.

When mothers discouraged using general directives, the message only affected 12-month-old experienced crawlers at the safe and borderline drop-offs where they increased their tendency to avoid but did not change their use of alternative descent strategies. Mothers’ discouraging general directives led 18-month-olds to a different interpretation of the message as compared to the encouraging condition on borderline and risky drop-offs. When encouraged, 18-month-olds implemented the message by sliding down, not walking. But when discouraged, they stayed put and avoided rather than slid. In fact, avoidance tripled and alternative strategies halved from encouraging to discouraging conditions.

### Effects of Social Information on Novice Infants’ Motor Decisions

Despite the perceptual differences between drop-off and slopes, 12-month-old novice walkers were still reckless on risky drop-offs. They attempted to walk on the risky drop-offs on 55% of encouraging trials and on the 90-cm cliff on 45% of encouraging trials. Recklessness was not limited to one or two infants; 6 of 10 novice walkers attempted to walk over the 90-cm drop-off paralleling the 63% of infants who walked over the 90-cm drop-off in the Kretch and Adolph (2013) study. As expected, experienced 12-month-old crawlers and 18-month-old walkers rarely attempted to crawl or walk at those increments.

Remarkably, mothers’ discouragement did not prevent novice 12-month-old infants from walking on risky drop-offs. Novice 12-month-old walkers attempted to walk on risky drop-offs on 64% of discouraging trials and on the 90-cm cliff on 30% of discouraging trials. In contrast, experienced 12-month-old crawlers never attempted to crawl on risky drop-offs and were more likely to remain on the starting platform when faced with a 90-cm cliff. One possible explanation is that walkers are used to falling and are not averse to frequent falls. However, this is not the case. Longitudinal observations of infants descending slopes show that falls decrease with weeks of walking experience (Adolph, 1997) and when tested in a falling paradigm, walking infants show negative affect after frequent falls (Joh and Adolph, 2006). Another explanation is that novice walkers plunge over the edge of impossibly large drop-offs because they lack the locomotor experience to recognize the potential risk, and they fail to recognize that they don’t know what to do. If infants did recognize that they don’t know, then they should use social information on all drop-offs. Again, this was not so for the novice 12-month-olds. In contrast, experienced 18-month-old walkers in slippery Teflon-soled shoes recognized that slopes that were typically manageable (when tested barefoot or in rubber-soled shoes) were now impossible and updated their use of social information to a larger range of slopes, even the most shallow ones (Adolph et al., 2011). Manipulations of social information contribute in unique ways to an understanding of how locomotor experience affects infants’ use of social information. Apparently, 12-month-old novice walkers face a double whammy; they do not perceive that extreme increments pose a potential threat of falling and they fail to recognize the value of social information.

## Conclusion

Findings from the current study provide new insights into the role of locomotor experience in infants’ integration and use of perceptual and social information at the brink of a drop-off. Although social information affects infants’ motor decisions, it is not the sole authority. Experienced crawlers and walkers selectively defer to social information when perceptual information is ambiguous. In contrast, novice walkers, who have a high tendency to walk regardless of risk, take mothers’ advice only at extreme drop-offs and even then, do so inconsistently. Over the course of development, as infants are figuring out what they can do by themselves, they are also figuring out the relevance of others as sources of information.

## Author Contributions

LBK helped with study design, coded, analyzed, and wrote the manuscript; CTL designed the study, conceptualized and helped to write the manuscript, and provided the funding for this research; KEA designed the study, conceptualized and helped to write the manuscript, and provided the funding for this research.

## Conflict of Interest Statement

The authors declare that the research was conducted in the absence of any commercial or financial relationships that could be construed as a potential conflict of interest.

## Footnotes

1 www.datavyu.org
